# Abnormal plasma ceramides refine high-risk patients with worsening heart failure

**DOI:** 10.3389/fcvm.2023.1185595

**Published:** 2023-06-29

**Authors:** Lu Ren, Fengjuan Li, Xin Tan, Yangkai Fan, Bingbing Ke, Yixin Zhang, Hongfeng Jiang, Lixin Jia, Yuan Wang, Jie Du

**Affiliations:** ^1^ Beijing Collaborative Innovation Centre for Cardiovascular Disorders, The Key Laboratory of Remodeling-Related Cardiovascular Disease, Ministry of Education, Beijing Anzhen Hospital, Capital Medical University, Beijing, China; ^2^ Beijing lnstitute of Heart, Lung, and Blood Vessel Diseases, Beijing Anzhen Hospital, Capital Medical University, Beijing, China; ^3^ Department of Cardiology, Cardiovascular Center, Beijing Friendship Hospital, Capital Medical University, Beijing, China; ^4^ Institute for Cardiovascular Prevention, Ludwig-Maximilians-Universitat Munchen (LMU), Munich, Germany; ^5^ Department of Cardiology, Beijing Anzhen Hospital, Capital Medical University, Beijing, China

**Keywords:** worsening heart failure, ADHERE score, biomarker, organ dysfunction, prognosis

## Abstract

**Background:**

Worsening heart failure (WHF) is a heterogeneous clinical syndrome with poor prognosis. More effective risk stratification tools are required to identify high-risk patients. Evidence suggest that aberrant ceramide accumulation can be affected by heart failure risk factors and as a driver of tissue damage. We hypothesized that specific ceramide lengths and ratios serve as biomarkers for risk stratification in WHF patients by reflecting pathological changes of distinct organ dysfunctions.

**Medthods:**

We measured seven plasma ceramides using liquid chromatography-mass spectrometry (LC-MS) in 1,558 patients, including 1,262 participants in retrospective discovery set and 296 WHF patients in prospective validation set in BIOMS-HF study (Registry Study of Biomarkers in Heart Failure). Univariable and multivariable logistic regression models were constructed to identify associations of ceramides with organ dysfunctions.

**Results:**

We constructed three ceramide-based scores linked independently to heart, liver, and kidney dysfunction, with ceramides and ratios included in each score specifying systemic inflammation, chronic metabolic disorder, and water-sodium retention. The combined ceramide heart failure score (CHFS) was independently associated with adverse outcomes [Hazard Ratio, 2.80 (95% CI: 1.78–4.40; *P* < 0.001); 2.68 995% CI: 1.12–6.46; *P* = 0.028)] and improved the predictive value of Acute Decompensated Heart Failure National Registry score and BNP [net reclassification index, 0.34 (95% confidence interval, CI: 0.19–0.50); 0.42 (95% CI: 0.13–0.70)] in the discovery and validation set, respectively. Lower BNP levels, but higher CHFS had the highest hazard of future adverse events in WHF patients.

**Conclusion:**

Abnormal plasma ceramides, associated with heart and peripheral organ dysfunctions, provide incremental prognostic information over the ADHERE score and brain natriuretic peptide concentration for risk stratification in WHF patients. This may facilitate the reclassification of high-risk patients in need of aggressive therapeutic interventions.

## 1. Introduction

Worsening heart failure (WHF) is a life-threatening disorder with a 1-year event rate of 40% (1). The current treatment for WHF is mostly symptomatic; at best, it is tailored according to the initial hemodynamic status. However, some patients experience high mortality and hospital readmission rates (2).

Patients with WHF often have coexisting multi-organ injury/dysfunction (heart, kidney, and liver) upon admission (3–6). Maladaptive crosstalk between the heart and injured peripheral organs leads to insufficient peripheral perfusion, a persistent congestive state, and abnormal changes in cardiac systolic or diastolic function, resulting in pathological ventricular remodeling and adverse outcomes (7). Dysfunction or injury to the peripheral organs is a common result of different etiological and risk factors, as suggested by the severity and complex pathological heterogeneity of WHF (8). Several clinical markers have been recommended in the European Heart Failure (HF) guidelines for the identification of organ dysfunction, including alanine transaminase (ALT), aspartate transaminase (AST), creatine kinase-MB, and estimated glomerular filtration rate (9). The natriuretic peptide concentration and Acute Decompensated Heart Failure National Registry (ADHERE) score, which are derived from the patient's blood urea nitrogen concentration, systolic blood pressure, and creatinine concentration, provide a means of assessing an individual patient's risk of inpatient mortality due to HF (10–12). Despite the availability of these measures, we have not been able to reduce adverse clinical outcomes in patients with WHF. Therefore, identifying the underlying pathological changes linked to different types of organ dysfunction could help improve the risk stratification and treatment of patients with WHF.

Various underlying pathophysiological changes, such as proinflammatory activation, oxidative stress, persistent ischemia, unresolved hyperemia, unhealthy metabolic status, and hypoperfusion, often coexist in patients with WHF who have organ injury/dysfunction (13). Several studies have shown that lipid metabolism reflects the underlying pathological processes in peripheral organs and aggravates the deterioration of cardiac function (14–16). The bioactive sphingolipid ceramide is both a structural component of the cell membrane and a signaling molecule that regulates endoplasmic reticulum stress, apoptosis, mitochondrial energy metabolism, and insulin resistance (17). Evidence suggests that distinct ceramides are closely related to several cardiometabolic diseases such as diabetes, hypertension, and coronary heart disease, and act as prognostic biomarkers for cardiovascular diseases (14, 18–20). A recent study conducted using an animal model of fatty liver disease showed that plasma Cer(d18:1/24:1) and Cer(d18:1/24:1) levels were both affected by the activation of ceramide synthase 2, which strongly indicates metabolic disorders in the liver tissue. Ahmad et al. showed that tissue-specific ceramide synthase 6 in glomerular podocytes produces Cer(d18:1/16:0), which affects renal perfusion by participating in oxidative stress and inflammatory reactions during acute/chronic kidney injury (21). Thus, it is becoming evident that distinct plasma ceramides are probably tissue-specific and have different physiological functions. Exploring the relationship between distinct organ dysfunctions and specific ceramide lengths in patients with WHF may provide evidence to further elucidate how these bioactive sphingolipids affect the occurrence and development of HF. Therefore, we hypothesized that specific ceramide lengths and ratios could serve as biomarkers for risk stratification in patients with WHF by reflecting pathological changes in distinct organ dysfunction.

In the present study, we identified a correlation between distinct ceramides and adverse outcomes in patients with WHF. We assessed the association between ceramide molecules and organ dysfunction. Finally, we established a ceramide heart failure score (CHFS) and verified its feasibility for risk stratification to provide greater insight into the potential role of ceramide in WHF.

## 2. Methods

### 2.1. Study population

The overall study design is shown in Supplementary Figure S1. This study comprised a cross-sectional discovery set, a retrospective discovery set, and a prospective validation cohort. Data supporting the findings of this study are available from the corresponding author upon reasonable request.

One cross-sectional study was established to evaluate whether plasma ceramides are associated with heart failure progression. The cross-sectional set (Supplementary Figure S1A) included 447 patients in 3 heart failure stages: healthy controls, preclinical patients, and patients with worsening heart failure. All the participants were age- and sex-matched. Patients with worsening heart failure were identified from the Registry Study of Biomarkers in Heart Failure (BIOMS-HF) cohort (22). The inclusion criteria were as follows: A total of 149 healthy controls were recruited from the population participating in the physical examination center at Anzhen Hospital. The inclusion criterion was a healthy clinical status with a potential risk of HF but without structural heart disease. The 149 participants with preclinical HF had a history of structural heart disease but no symptoms of HF.

Discovery and validation sets were obtained from BIOMS-HF, a retrospective and prospective cohort study (Supplementary Figure S1B). The retrospective discovery cohort included 964 patients with WHF who visited the Beijing Anzhen Hospital, Capital Medical University, between August 2017 and March 2019 (BIOMS-HF study registration number: NCT03784833 in ClinicalTrials.gov). The prospective validation set included 296 patients with WHF who visited Beijing Anzhen Hospital, Capital Medical University, between April 2019 and June 2019. This study was based on the inclusion criteria of BIOMS-HF: age of ≥18 years, HF symptoms and signs (dyspnea or minimal exertion at rest, dry and wet oral, pleural and ascites, peripheral edema, or pulmonary congestion on x-ray films), and brain natriuretic peptide (BNP) and N-terminal pro-BNP (NT-pro-BNP) concentrations of ≥35 and ≥125 pg/ml, respectively (23). For a diagnosis of WHF, patients were required to have a relevant history of HF symptoms and signs and a diagnosis of structural heart disease. Structural heart disease included any one of the following criteria: an increased left ventricular end-diastolic size as measured by echocardiography (≥55 mm); a left ventricular ejection fraction (LVEF) of ≤50%; ventricular septal thickening (>12 mm) or left ventricular posterior wall thickening (>12 mm) as measured by echocardiography; severe valve stenosis/dysfunction; or significant myocardial abnormality (cardiomyopathy), congenital heart disease, or previous cardiac surgery (24). The detailed study design is presented in Supplementary Figure S1B. A total of 964 patients in the discovery set and 296 in the validation set were analyzed, including 972 patients without composite events and 288 patients with composite events.

This study was approved by the local ethics committee. The study was performed in accordance with the requirements of the Declaration of Helsinki. All the participants provided written informed consent.

### 2.2. Follow-up and outcomes

The primary endpoints were all-cause mortality and the following events: repeated hospitalization for HF, recommendation for heart transplantation by physicians, implantation of a cardiac resynchronization therapy defibrillator (25), and follow-up with a New York Heart Association functional classification of IV. Unplanned emergency visits or hospitalizations leading to HF deterioration were defined as those due to HF. Data on events were obtained from telephone or electronic medical records. The primary endpoints and adverse events were reviewed and confirmed by certified physicians to ensure accuracy. There were 315 (33%) patients who suffered from HF event/hospitalization, and 152 (16%) patients who died in the experimental period in the discovery set. The validation set included 97 (33%) patients with HF event/hospitalization and 41 (14%) patients died in the experimental period. During the median follow-up of 325.5 days in the discovery set, 129 patients were lost to follow-up (follow-up rate: 86.6%). During a median follow-up of 208 days in the validation set, 13 patients were lost to follow-up (follow-up rate: 95.6%).

### 2.3. Sample collection and quantification of ceramides

Fasting blood samples were collected in ethylenediaminetetraacetic acid tubes, centrifuged, aliquoted, and stored at −80°C until analysis. Ultra-performance liquid chromatography-tandem mass spectrometry was performed to quantitatively detect several plasma ceramides [Cer(d18:1/14:0), Cer(d18:1/16:0), Cer(d18:1/18:0), Cer(d18:1/24:0), Cer(d18:1/20:0), Cer(d18:1/22:0), Cer(d18:1/24:0), and Cer(d18:1/24:1)] using a Thermo TSQ Quantum mass spectrometer equipped with an electrospray ionization probe and interfaced with the Agilent 1,290 Infinity LC system (Agilent, Palo Alto, CA, United States). The injection volume of the extracted samples was 10 µl. The details of the ceramide tests were elaborated from previous research conducted by our team (26).

### 2.4. Definition of organ dysfunction/injury

Organ dysfunction/injury was defined based on baseline measurements. A troponin I concentration greater than the upper reference limit (URL) (>0.056 ng/ml) was considered myocardial injury (27). Renal insufficiency was defined as an estimated glomerular filtration rate of <60 ml/min/1.73 m^2^, calculated using the Modification of Diet in Renal Disease Study equation (28, 29). The presence of at least one of these abnormal liver function test results indicates liver injury or dysfunction: ALT and AST levels beyond the upper limit reference range for liver function (male: 9–50 U/L, female: 7–40 U/L; AST: male:15–40 U/L; female: 13–35 U/L), bilirubin concentration higher than the URL (>1.3 mg/ml), and albumin concentration lower than the lower reference limit (<3.5 mg/dl) (30, 31).

### 2.5. Statistical analysis

Continuous variables with a normal distribution are expressed as means with standard deviations. The statistical significance of the differences between the groups was tested using analysis of variance, Student's *t*-test, or the *χ*^2^ test, as appropriate. Statistical significance was set at *P* < 0.05. Logistic regression was used to estimate the odds ratios (OR) per standard deviation. Cox proportional hazards were used to calculate hazard ratios (HR). Proportional hazard assumptions were tested using Schoenfeld residuals. The Spearman correlation coefficient was used to evaluate association for continuous variables. Logistic regression analysis was used to evaluate the correlation between scores with categorical clinical outcomes (e.g., diabetes, ascites, ortopnea). Excluding cases with missing values may bias the results (32); thus, imputation proceeded in two steps: first, continuous variables were imputed using the EM algorithm to create a monotone missingness pattern, and then categorical variables were imputed using the logistic regression method. Statistical analyses were performed using Stata 15.0 statistical software and R.

Univariate and multivariate logistic regression models were constructed to determine the relationship between ceramide length and distinct organ dysfunction (heart, liver, and kidney). To assess the robustness of the final model, we performed a 1,000-repeat boot analysis (using Stata's “swboot” package). Variables selected more than 700 times were considered as robust predictors. The Hosmer–Lemeshow test was performed to test for the model's goodness-of-fit. A restricted cubic spline (three nodes of all variables) was constructed to display the relationship between the Ceramide lengths and ratios and organ injuries.

The ceramide heart, liver, and kidney scores were constructed based on the logistic model coefficient for each given ceramide concentration and the corresponding ratio:

$$\mathrm{ceramide}\;\mathrm{heart}/\mathrm{liver}/\mathrm{kidney}\;\mathrm{score}\;=\underset{n=k}{\sum }\beta k\times \mathrm{ceramide}\;\mathrm{or}\;\mathrm{ratio}\;\mathrm{concentration}+b$$

where *β*k is the coefficient of the multivariable logistic model for each ceramide length or ratio concentration and b is the constant term of the model. We decided ceramide heart, liver, and kidney scores after comparing models through the Akaike information criteria (AIC) and the Bayesian information criteria (BIC). Because dysfunction/injury to more than one organ (heart, kidney, or liver) is a well-recognized independent predictor of poor outcomes, we constructed the CHFS as the sum of the ceramide heart, liver, and kidney scores. A multicollinearity test was conducted to detect the multicollinearity between key variables of CHFS (ceramide heart score, ceramide liver score, and ceramide kidney score). We identified the effect modification on the BNP level using multiplicative interaction terms.

## 3. Results

### 3.1. Study population, plasma ceramide length, and ratio distributions in the discovery set

Among the 964 patients with WHF in the discovery set, the baseline characteristics of the patients with and without composite events are shown in Table 1. Patients with composite events were older and had a higher incidence of diabetes mellitus. During laboratory examinations, patients with composite events had higher BNP, lower high-density lipoprotein, higher high-sensitivity C-reactive protein, and higher creatine kinase concentrations. There were no other statistically significant differences in baseline characteristics. The characteristics of the validation set are presented in Supplementary Table S4. The baseline levels of Cer(d18:1/16:0), Cer(d18:1/18:0), Cer(d18:1/24:1), and Cer(d18:1/24:0) and the distinct ratios of Cer(d18:1/16:0)/Cer(d18:1/24:0), Cer(d18:1/18:0)/Cer(d18:1/24:0), and Cer(d18:1/24:1)/Cer(d18:1/24:0) were significantly different between event and no-event groups in the discovery cohort. No significant differences were found in other chain species [Cer(d18:1/14:0), Cer(d18:1/20:0), and Cer(d18:1/22:0)] (Supplementary Table S1). We also observed that the Cer(d18:1/16:0), Cer(d18:1/18:0), and Cer(d18:1/24:1) levels increased, whereas the Cer(d18:1/24:0) levels decreased. The results are presented in Supplementary Figure S2. The remaining chain lengths [Cer(d18:1/14:0), Cer(d18:1/20:0), and Cer(d18:1/22:0)] showed no trends or significant differences.

**Table 1:** Baseline characteristics of event and non-event groups of patients with worsening heart failure in the discovery cohort.

	No-event group (*N* = 732)	Event group (*N* = 232)	*P* value
Male sex, *n* (%)	613 (62)	176 (65)	0.283
Age, years	62 ± 14.4	68 ± 14.0	<0.001
Clinical history, *n* (%)			
Smoking	159 (23)	36 (18)	0.131
Hypertension	420 (59)	137 (60)	0.722
Diabetes mellitus	243 (34)	95 (42)	0.031
Hyperlipidemia	244 (34)	68 (30)	0.210
CKD	122 (17)	53 (23)	0.040
Prior MI	116 (16)	40 (18)	0.657
Prior stroke	98 (14)	34 (15)	0.628
Atrial fibrillation	207 (29)	71 (31)	0.486
VHD	278 (39)	79 (35)	0.264
CAD	391 (55)	127 (56)	0.835
NYHA			0.019
II	187 (28)	59 (27)	
III	324 (48)	88 (40)	
IV	158 (24)	72 (33)	
Hemodynamics			
SBP, mmHg	128 ± 24	125 ± 24	0.075
DBP, mmHg	76 ± 15	74 ± 17	0.099
Heart rate,bpm	84 ± 21	86 ± 20	0.055
LVEF, %	45 ± 15	44 ± 15	0.209
Laboratory data			
HDL-C, mmol/L	1.08 ± 0.58	1.00 ± 0.32	0.066
LDL-C, mmol/L	2.53 ± 0.92	2.47 ± 0.81	0.814
TG, mmol/L	1.35 ± 0.88	1.23 ± 1.08	0.052
TC, mmol/L	4.12 ± 1.12	4.00 ± 1.04	0.375
hs-CRP	8.12 ± 10.89	11.57 ± 13.57	<0.001
BNP	980 ± 1,022	1,414 ± 1,389	<0.001
D-Dimer	633.1 ± 2,397.5	954.3 ± 1,886.7	<0.001
HB, g/L	133.4 ± 23.5	127.2 ± 24.1	<0.001
cTNI	1.70 ± 7.17	2.78 ± 8.71	0.006
Na+	139.0 ± 3.87	138.0 ± 4.99	0.001
K+	4.19 ± 0.56	4.29 ± 0.71	0.091
LnCr	4.52 ± 0.58	4.62 ± 0.66	0.048
Therapy			
Statin	297 (51)	79 (54)	0.483
ACEI/ARB	205 (35)	42 (29)	0.145
β-blocker	353 (59)	87 (59)	0.887
Diuretic	393 (66)	107 (72)	0.134
Spironolactone	279 (47)	76 (52)	0.296
Digoxin	178 (24)	35 (27)	0.975
ANRI	71 (11)	11 (9)	0.414

LVEF, left ventricular ejection fraction; BNP, brain natriuretic peptide; CKD, chronic kidney disease; MI, myocardial infarction; VHD, valvular heart disease; CAD, coronary artery disease; NYHA, New York Heart Association; SBP, systolic blood pressure; DBP, diastolic blood pressure; HDL-C, high-density lipoprotein cholesterol; LDL-C, low-density lipoprotein cholesterol; TC, total cholesterol; TG, triglycerides; hs-CRP, high sensitivity C-reactive protein; HB, hemoglobin; CTNI, high-sensitivity cardiac troponin I; ACEl/ARB, angiotensin converting enzyme inhibitor or angiotensin receptor blocker; ANRI, enkephalinase inhibitors.

### 3.2. Plasma ceramides and ratios in patients with WHF with adverse clinical outcomes in the discovery set

**Figure 1:**
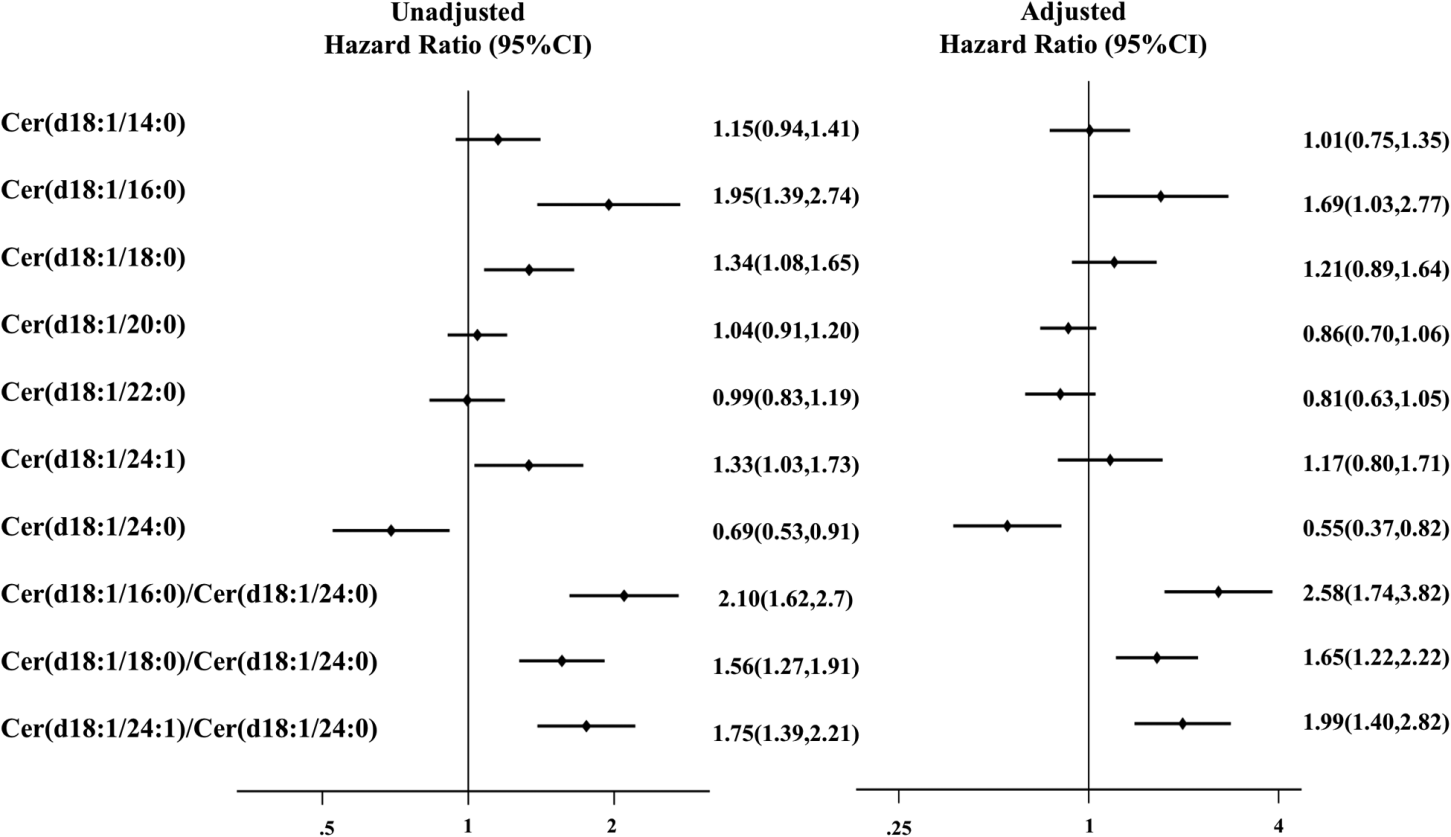
Association of combined endpoint with baseline ceramide lengths and ratios in the discovery cohort. Model was adjusted for sex, age, body mass index, systolic blood pressure, diastolic blood pressure, heart rate, diabetes, hypertension, hyperlipidemia, chronic renal disease, atrial fibrillation, coronary heart disease, and smoking. Effect size (odds ratio and 95% confidence interval) is presented for each ceramide length and ratio.

In the multivariate Cox regression analyses, Cer(d18:1/16:0), Cer(d18:1/18:0), Cer(d18:1/24:1), and Cer(d18:1/24:0) accumulation was positively associated with the primary endpoint after adjustment for age and sex; the HR was 1.85 [95% confidence interval (CI): 1.38–2.74], 1.34 (1.08–1.65), 1.33 (1.02–1.76), and 0.69 (0.53–0.91), respectively; Supplementary Table S2]. After adjusting for age, sex, and clinical risk factors (Model 1, including sex, age, body mass index, systolic blood pressure, diastolic blood pressure, heart rate, diabetes, hypertension, hyperlipidemia, chronic renal disease, atrial fibrillation, coronary heart disease, and smoking), the ratios Cer(d18:1/16:0)/Cer(d18:1/24:0), Cer(d18:1/18:0)/Cer(d18:1/24:0), and Cer(d18:1/24:1)/Cer(d18:1/24:0) were still significantly related to adverse events [HR: 2.58 (95% CI: 1.74–3.82); HR: 1.65 (95% CI: 1.22–2.22); and HR: 1.99 (95% CI: 1.40–2.82), respectively] (Figure 1).

### 3.3. Ceramide lengths and ratios in patients with heart, liver, and kidney dysfunction in the discovery set

Univariate and multivariate logistic regression analyses of ceramide lengths and ratios for different organ dysfunctions in the discovery cohort are shown in Table 2. Increases of Cer(d18:1/18:0) and the ceramide ratios Cer(d18:1/18:0)/Cer(d18:1/24:0) were correlated with cardiac injury; very-long-chain ceramides [Cer(d18:1/24:0) and Cer(d18:1/24:1)] and Cer(d18:1/16:0) were correlated with liver dysfunction; Cer(d18:1/16:0) and Cer(d18:1/16:0)/Cer(d18:1/24:0) were correlated with renal dysfunction (all *P* < 0.005). Before the detailed analysis, we quantified the appropriateness of our statistical models using AIC and BIC (Supplementary Table S7). The final model was selected considering both the statistical significance and Bayesian information criteria and detailed equation was shown in Supplementary Table S10.

**Table 2:** Univariate and multivariate logistic regression of ceramide lengths and ratios for different organ dysfunctions in the discovery cohort.

Organ dysfunctions	Univariable			Multivariable		
	Odds ratio (95% CI)	*z*-value	*P* value	Odds ratio (95% CI)	*z*-value	*P* value
Cardiac injury						
Cer(d18:1/16:0)	3.45 (2.27–5.25)	5.78	<0.001			
Cer(d18:1/18:0)	2.45 (1.88–3.22)	6.57	<0.001	1.91 (1.29–2.83)	3.22	0.001
Cer(d18:1/24:1)	2.45 (1.76–3.42)	5.27	<0.001			
Cer(d18:1/24:0)	1.67 (1.21–2.32)	3.11	0.002			
Cer(d18:1/16:0)/Cer(d18:1/24:0)	1.33 (0.98–1.82)	1.80	0.072			
Cer(d18:1/18:0)/Cer(d18:1/24:0)	1.67 (1.32–2.11)	4.29	<0.001	2.20 (1.51–3.21)	4.08	<0.001
Cer(d18:1/24:1)/Cer(d18:1/24:0)	1.51 (1.08–2.10)	1.44	0.015			
Liver dysfunction						
Cer(d18:1/16:0)	2.43 (1.71–3.47)	4.92	<0.001	2.08 (1.24–3.51)	2.76	0.006
Cer(d18:1/18:0)	1.59 (1.27–1.97)	1.04	<0.001			
Cer(d18:1/24:1)	2.03 (1.54–2.69)	4.97	<0.001	2.28 (1.51–3.45)	3.90	<0.001
Cer(d18:1/24:0)	0.56 (0.63–0.79)	−4.17	<0.001	0.46 (0.32–0.65)	−4.41	<0.001
Cer(d18:1/16:0)/Cer(d18:1/24:0)	2.03 (1.53–2.70)	4.91	<0.001			
Cer(d18:1/18:0)/Cer(d18:1/24:0)	1.63 (1.32–2.01)	4.67	<0.001			
Cer(d18:1/24:1)/Cer(d18:1/24:0)	2.46 (1.83–3.31)	6.00	<0.001	2.07 (1.38–3.10)	3.51	<0.001
Kidney dysfunction						
Cer(d18:1/16:0)	2.68 (1.89–3.79)	5.54	<0.001	3.10 (1.73–5.56)	3.79	<0.001
Cer(d18:1/18:0)	1.33 (1.08–1.63)	2.69	0.007			
Cer(d18:1/24:1)	1.72 (1.31–2.24)	3.94	<0.001			
Cer(d18:1/24:0)	1.27 (0.96–1.68)	1.70	0.088			
Cer(d18:1/16:0)/Cer(d18:1/24:0)	1.50 (1.15–1.97)	2.96	0.003	1.64 (1.08–2.48)	2.34	0.019
Cer(d18:1/18:0)/Cer(d18:1/24:0)	1.15 (0.94–1.39)	1.40	0.161			
Cer(d18:1/24:1)/Cer(d18:1/24:0)	1.40 (1.06–1.83)	1.72	0.016			

### 3.4. Ceramide cardiac/liver/kidney scores and clinical characteristics in the discovery set

**Figure 2:**
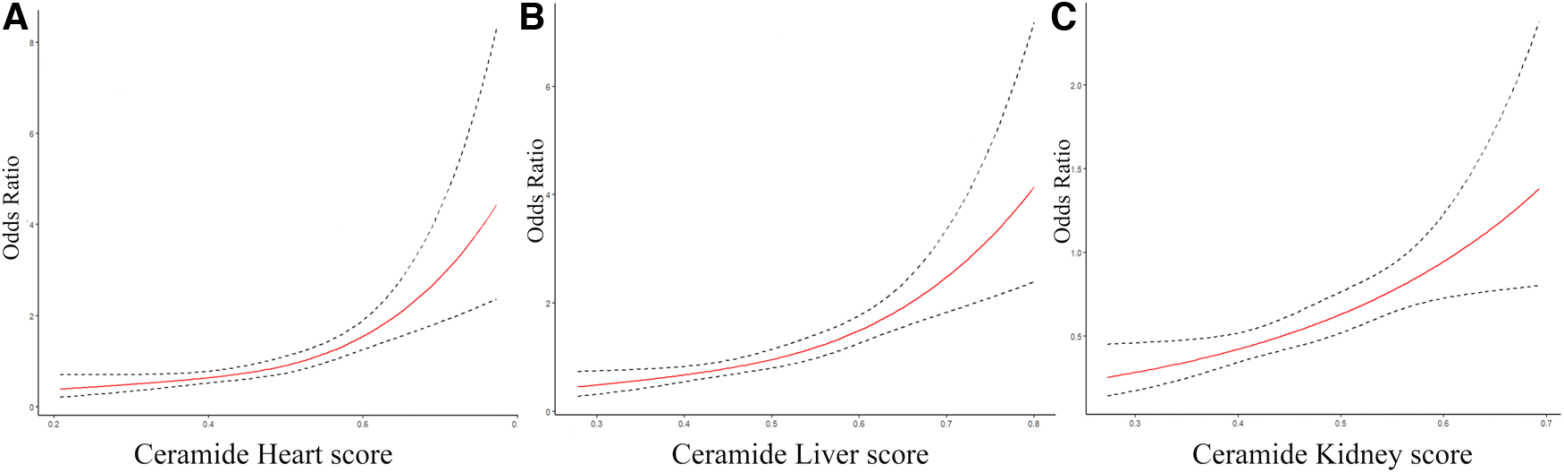
The restricted cubic spline of ceramide heart, liver, and kidney scores for different organ dysfunctions. (**A**) Restricted cubic spline of the association between the ceramide heart score and the cardiac injury. (**B**) Restricted cubic spline of the association between the ceramide liver score and liver dysfunction. (**C**) Restricted cubic spline of the relationship between the ceramide kidney score and kidney dysfunction. The solid lines indicate estimates of the odds ratios of organ damage across continuous levels of different ceramide scores, fitted using logistic regression analysis. The dashed lines indicate the 95% confidence intervals.

**Figure 3:**
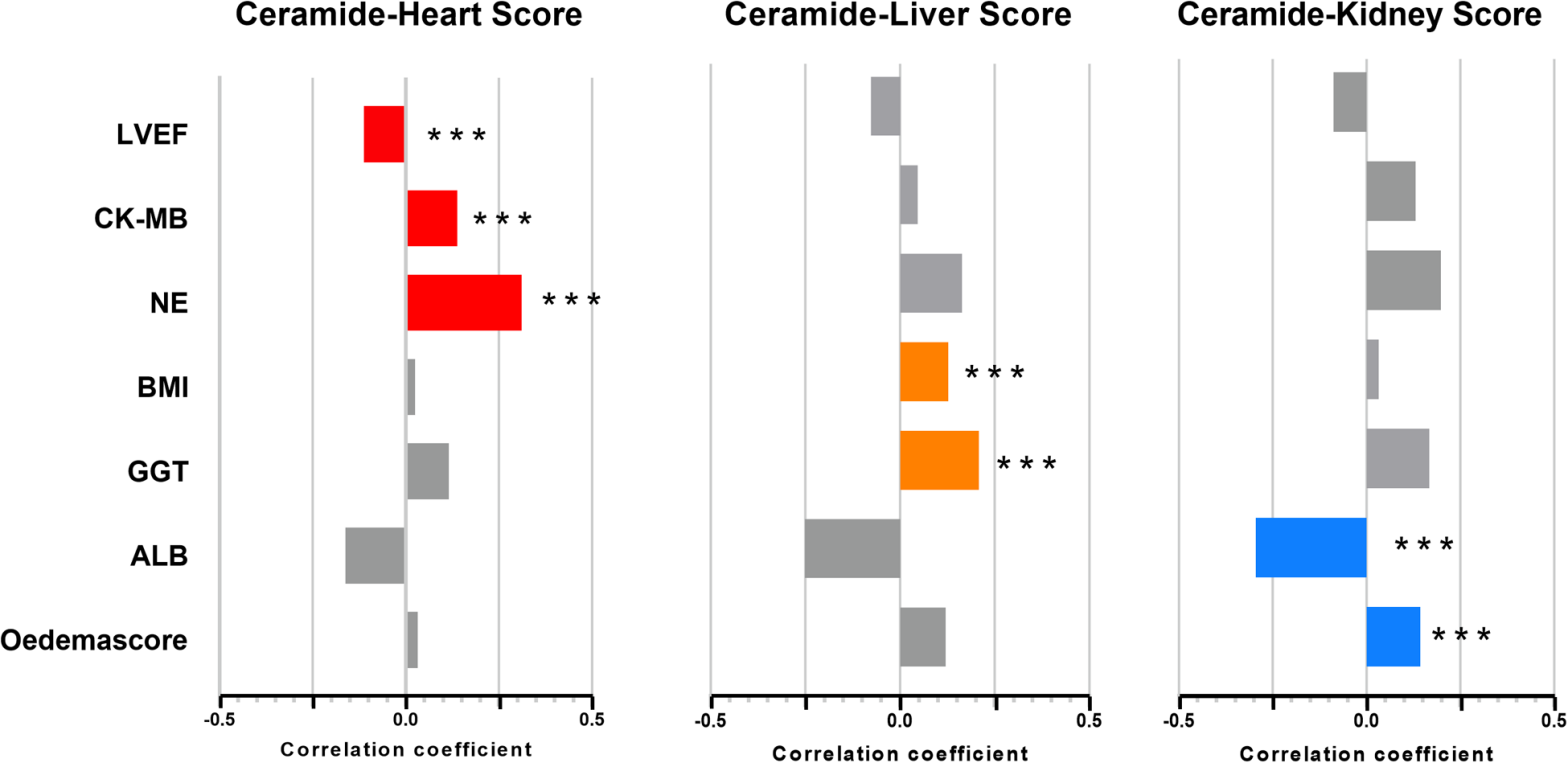
Association of ceramide cardiac injury/liver dysfunction and kidney dysfunction scores and clinical characteristics. DM, diabetes mellitus; LVEF, left ventricular ejection fraction; NE, neutrophilic granulocytes; WBC, white blood cells. *P < 0.05, **P < 0.01, ***P < 0.001.

Furthermore, we constructed ceramide heart, liver, and kidney scores based on plasma ceramide concentrations and distinct ratios. The restricted cubic spline of ceramide levels, cardiac injury/liver dysfunction, and kidney dysfunction scores are shown in Figure 2. The Hosmer–Lemeshow goodness-of-fit test for three logistic regression models shows that the models are acceptable (ceramide heart score: *P* = 0.123; ceramide liver score: *P* = 0.859; ceramide kidney score: *P* = 0.136) (Supplementary Table S6). We examined the correlations between these scores and the clinical characteristics in the discovery cohort of patients with WHF (Figure 3, Supplementary Figure S4). We found that the ceramide liver score was correlated with chronic metabolic injuries, including body mass index (BMI), glutamyl-transpeptidase (GGT), diabetes, orthopnea, and ascites; The ceramide kidney score significantly correlated with kidney function impairment and signs of water and sodium retention (edema score and orthopnea). Ceramide heart score associated with LVEF and cardiac tissue damage marker CKMB, suggesting a clinical phenotype related to myocardial damage. Our results demonstrated different patterns of clinical characteristics among the ceramide heart, liver, and kidney scores.

### 3.5. Performance of CHFS in both discovery and validation set

**Figure 4:**
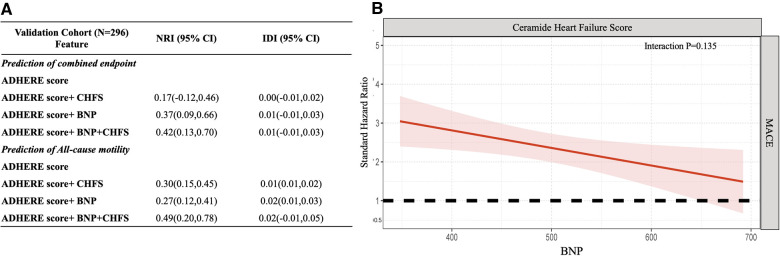
Abnormal ceramides from organ dysfunction indicate the greatest risk in worsening heart failure. (**A**) Additional value of CHFS for prediction of combined endpoint and all-cause motility in the validation cohort. (**B**) The standard hazard for ceramide heart failure score across BNP levels for the composite endpoint in worsening heart failure patients. This figure demonstrates that the hazard of CHFS is higher in lower BNP worsening heart failure patients. ADHERE predictors: age, sex, urea, heart rate, and systolic blood pressure. CHFS consists of the combined ceramide heart, liver, and kidney scores. Point estimates and confidence limits for reclassification (NRI) and fit statistics are described in the Methods section. ADHERE, Acute Decompensated Heart Failure National Registry; BNP, brain natriuretic peptide; CHFS, ceramide heart failure score; CI, confidence interval; NRI, net reclassification index; WHF, worsening heart failure.

Finally, we developed a novel CHFS comprising the ceramide heart, liver, and kidney scores to model the clinical use of ceramide in patients with WHF. Test of multicollinearity for all variables resulted in the variance inflation factor (VIF) scores ranging from 1.07 to 2.19, indicating no concerns about multicollinearity (Supplementary Table S7). The CHFS was independently correlated with all-cause mortality in both the discovery set and validation cohort (HR: 2.76; 95% CI: 1.75–4.33; *P* < 0.001; HR: 3.47; 95% CI: 1.36–8.87; *P* = 0.009) and composite events (HR: 2.91; 95% CI: 1.23–6.88; *P* < 0.001; HR: 3.45; 95% CI: 1.10–10.80; *P* = 0.034) (Supplementary Table S3). We tested assumptions of proportional hazards using Schoenfeld residuals in both discovery and validation set (Supplementary Table S8). The global test showed no deviation from proportionality (discovery set: *P* = 0.329, validation set: *P* = 0.264) (Supplementary Table S9). The effect of the addition of CHFS to the ADHERE score (age, sex, urea concentration, heart rate, and systolic blood pressure) in predicting the composite endpoint or all-cause mortality was assessed using the category-free (continuous) net reclassification index (NRI) (Supplementary Table S4). We examined whether the addition of CHFS to the ADHERE logistic regression variables improved the overall WHF patient classification for all-cause mortality in the entire cohort (NRI: 0.31; 95% CI: 0.18–0.44). The ceramide heart failure score provided favorable risk reclassification for combined endpoint and all-cause motility beyond traditional ADHERE and BNP levels [net reclassification improvement, 0.42 (95% CI: 0.13–0.70) and 0.49 (95% CI: 0.20–0.78), respectively]. We observed no interaction between the CHFS and BNP levels in association with the composite event (interaction, *P* = 0.135, Figure 4). The hazard of the composite event for higher CHFS appeared to be greatest in patients with lower BNP levels and worsening heart failure in the validation set.

### 3.6. Subgroup analysis of CHFS performance in total cohort

We stratified worsening heart failure patients by LV ejection fraction and (Supplementary Table S5). After adjusting for clinical variables, CHFS were significantly associated with poor outcomes in patients with HFpEF [hazard ratio, 3.85 95% CI: 1.50–9.87; *P* = 0.005].

## 4. Discussion

In this study, we found that specific ceramide forms [Cer(d18:1/16:0), Cer(d18:1/18:0), Cer(d18:1/24:1), and Cer(d18:1/24:0)] were associated with HF severity. We then discovered a relationship between abnormal ceramide lengths and ratios and the risk of adverse events that subsequently progressed to refractory HF or death in patients with WHF in the discovery set. After adjusting for clinical risk factors, the ceramide ratios remained significantly associated with adverse events. Furthermore, we determined the ceramide heart, liver, and kidney scores with C18:0 and C18:0/Cer(d18:1/24:0), Cer(d18:1/16:0) and Cer(d18:1/24:1)/Cer(d18:1/24:0), and Cer(d18:1/16:0) and Cer(d18:1/16:0)/Cer(d18:1/24:0), respectively. Finally, we established that the CHFS (the summary of the three ceramide organ scores mentioned above) can be used as a risk stratification tool to identify high-risk patients with WHF. Abnormal plasma ceramide levels and ratios provide additional information about the injury/dysfunction of the heart and peripheral organs and the risk of HF deterioration, which may indicate specific pathophysiological characteristics of patients with WHF (33).

### 4.1. Myocardial ischemia, inflammation, and ceramide heart score

Studies have shown that ceramide destroys mitochondrial respiration and leads to the decompensation of mitochondrial function, thus affecting cardiac function (34). Inflammation is an important factor in the development of diastolic dysfunction in patients with WHF. Our data suggest that Cer(d18:1/18:0) and the ceramide ratio Cer(d18:1/16:0)/Cer(d18:1/24:0) are strongly correlated with myocardial ischemia and inflammation status. Studies have shown that CerS4 is expressed in the heart, has a high affinity for C18 and C20 acyl-CoA, and contributes to heart failure (35). In cardiomyocytes, CerS2 overexpression causes oxidative stress and mitochondrial dysfunction via lipid overload, eventually leading to apoptosis (36).

### 4.2. Water sodium retention symptoms with ceramide kidney score

In patients with worsening heart failure, myocardial ischemia and hypoxia cause a serious decline in cardiac output, leading to a decrease in circulating blood volume and poor perfusion of major peripheral organs. The imbalance of metabolic pathways, such as the inflammatory response, insulin resistance, weakening of anabolism, and accumulation of oxygen free radicals caused by abnormal hemodynamic changes, simultaneously affects cardiac function and aggravates disease progression (34). Studies revealed that under acute kidney damage state, production of reactive oxygen species and proximal tubular cell death induced activation of CerS6, causing the elevation of C16:0) both in kidney tissue and plasma (37–39). Our data suggest that Cer(d18:1/16:0) and Cer(d18:1/18:0) levels are associated with kidney damage.

### 4.3. Chronic metabolic vulnerability of liver and ceramide liver score

Previous studies have pointed out that the difference between long-chain (C16:0-C18:0) and ultra-long-chain (C20:0-C24:0) ceramide is produced by different ceramide synthase isomers (CerS1/5/6 and CerS2/4, respectively). Different concentrations of plasma ceramides may also reflect changes in the composition and function of cellular membranes within tissues. Our study showed that Cer(d18:1/16:0) and ceramide ratio Cer(d18:1/24:1)/Cer(d18:1/24:0) indicated chronic metabolic vulnerability and liver injury. By reducing ER stress and PEPCK expression, plasma very-long-chain ceramide (C24, C24:1) secreted by CerS2 reduced cellular stress and glucose homeostasis (40–42).

CerS isoforms may partially explain the altered ceramide composition, providing a biological explanation for the ceramide ratios. Studies have confirmed that *de novo* ceramide levels must be within a narrow range to maintain normal cardiac homeostasis. Thus, several hypothesized biological mechanisms link different ceramide and their ratios to cardiac dysfunction and increased mortality. (1) Ceramide metabolism and apoptosis in cardiac dysfunction: De Paola et al. showed that Cer(d18:1/16:0) generates reactive oxygen species and contributes to cardiomyocyte apoptosis. Interestingly, Cer(d18:1/24:0) counteracts Cer(d18:1/16:0)-mediated cytochrome c release in a dose-dependent manner, possibly by interfering with mitochondrial outer membrane channel formation and reducing membrane permeability (36, 43). (2) Ceramide promotes inflammatory activation during cardiac dysfunction: studies have revealed that ceramides contribute to inflammatory processes both as regulators of cytokine production and downstream effectors that mediate cytokine-induced stress responses (44–46). Ceramides can also induce vascular dysfunction by deactivating endothelial nitric oxide synthase (47). (3) Ceramides as potential inducers of fibrosis: ceramides stimulate the proteolytic processing of cAMP-responsive element-binding protein 3-like protein 1 (CREB3L1), a transcription factor that induces collagen production (48, 49). Although these actions have not been explored in the heart, these and other mechanisms may explain how ceramides contribute to myocardial energy metabolism, inflammation, and fibrotic responses that drive HF progression.

## 5. Conclusion

In line with these previous findings, our clinical results supported that the plasma ceramide length and ratio reflected the impairment of heart/liver/kidney organ function in WHF patients and indicated myocardial injury, inflammation, and water sodium retention. Abnormal plasma ceramides are associated with an increased risk of disease progression. Our study proved that CHFS is a practical tool for refining the risk stratification of a worsening adverse prognosis in patients with WHF. Further research is needed to discern the potential of specific ceramides to serve as therapeutic targets for HF-protective drugs.

### 5.1. Limitations

This study has several limitations. Because the study was retrospective and involved a single-center cohort, it may have been subjected to bias owing to uncontrolled confounding factors. We cannot prove a causal relationship between the duration of ceramide use and progression to end-stage HF and death. Moreover, ceramide is a sphingolipid derived from diet and other factors. Despite these limitations, our results provide new insights into the value of ceramides in evaluating the prognosis of patients with WHF.

## Data Availability

The raw data supporting the conclusions of this article will be made available by the authors, without undue reservation.
