# Supplementary material for: Abnormal plasma ceramides refine high-risk patients with worsening heart failure
Source: Front Cardiovasc Med. 2023 Jun 29;10:1185595. doi: 10.3389/fcvm.2023.1185595 (PMC10339027; doi:10.3389/fcvm.2023.1185595)
Supplement: Supplementary file 1 [file Table1.docx]

Supplementary Material

Abnormal plasma ceramides refine high-risk patients with worsening heart failure

Lu Ren, Yuan Wang*, Jie Du*

*** Correspondence:** Corresponding Author: [wangyuan980510@163.com](mailto:wangyuan980510@163.com)；jiedu@ccmu.edu.cn

## Supplementary Figures

**Supplementary Figure 1. Overall Study flow**


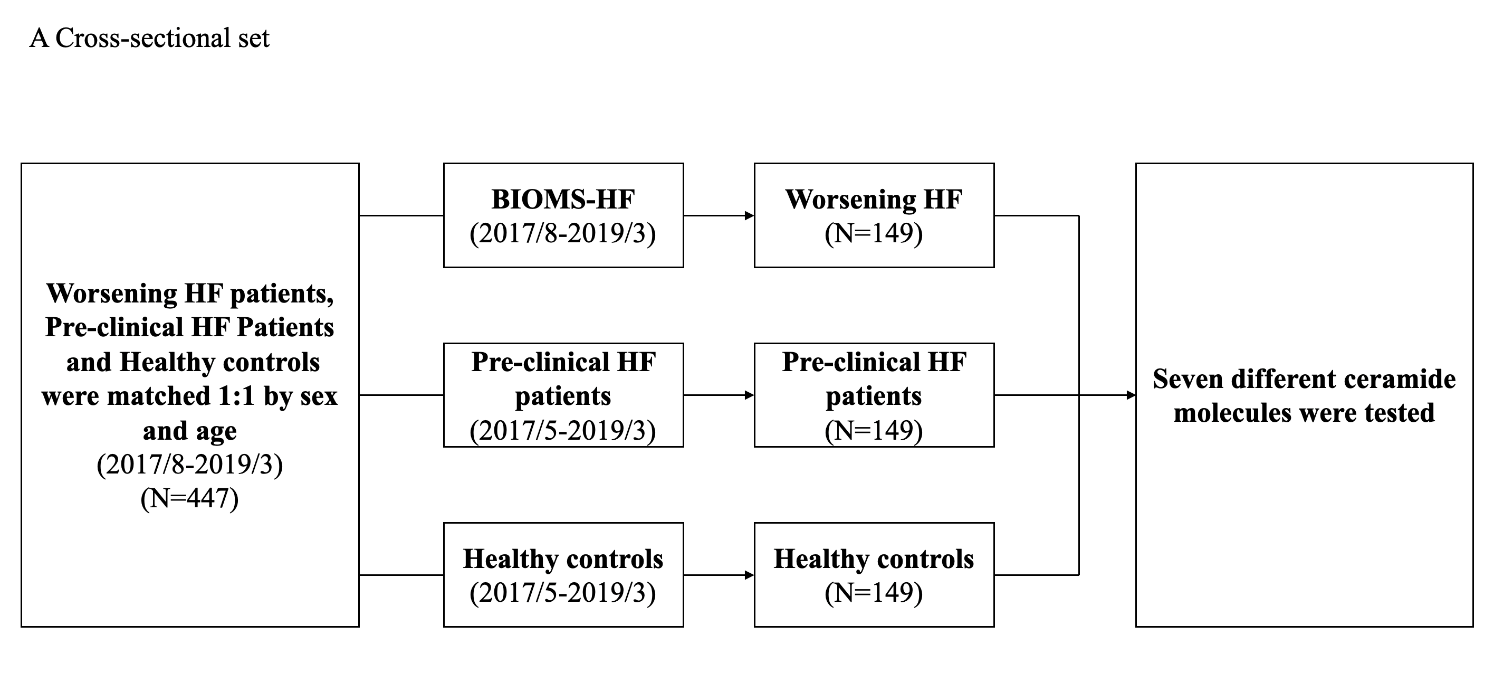
**A. Flow chart of cross-sectional study**

**Supplementary Figure 1b. Flow chart of discovery and validation set**


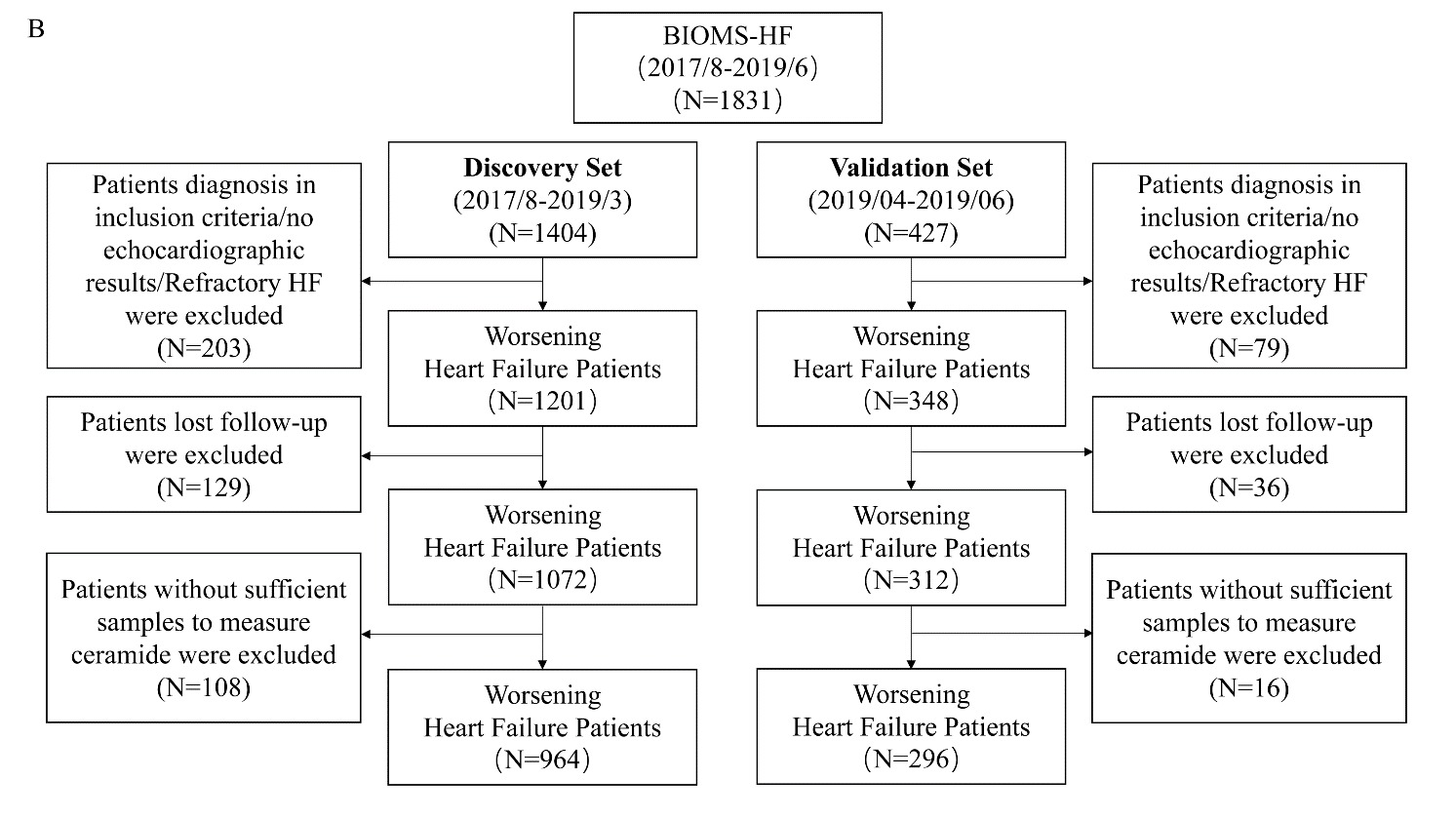


**Supplementary Figure 2. Distribution of ceramide length according to heart failure disease status.** P values of <0.05 were significant. P values were calculated according to the natural logarithm of the original value. The three patient groups were the healthy population, patients with preclinical heart failure, and patients with worsening heart failure.
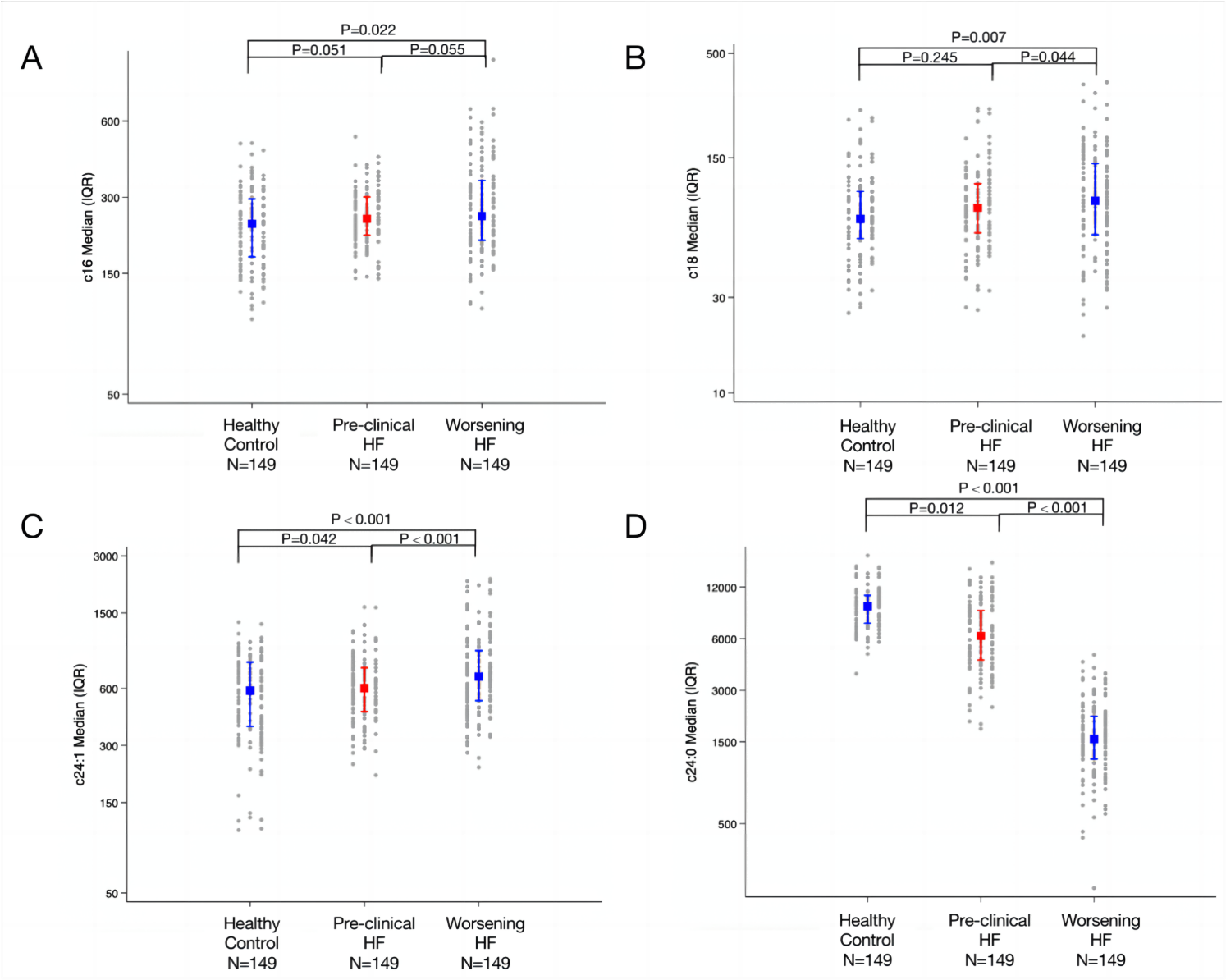


**Supplementary Table 1. Baseline differences in ceramide lengths and ratios in patients with worsening heart failure in discovery set.**

| Ceramide Type* | No-event Group  (N=732) | Event Group  (N=232) | P value* |
| --- | --- | --- | --- |
| Ceramide(d18:1/14:0), nmol/L | 5.4±3.93 | 5.9±4.12 | 0.137 |
| Ceramide(d18:1/16:0), nmol/L | 283.4±114.4 | 314.7±123.0 | ＜0.001 |
| Ceramide(d18:1/18:0), nmol/L | 88.2±62.6 | 96.1±64.0 | 0.047 |
| Ceramide(d18:1/20:0), nmol/L | 23.1±18.1 | 25.9±19.6 | 0.570 |
| Ceramide(d18:1/22:0), nmol/L | 459.0±319.1 | 489.2±340.9 | 0.835 |
| Ceramide(d18:1/24:1), nmol/L | 983.3±728.3 | 1074.3±567.0 | 0.035 |
| Ceramide(d18:1/24:0), nmol/L | 2027.9±928.5 | 1876.9±834.4 | 0.007 |
| Ceramide(d18:1/16:0)/Ceramide(d18:1/24:0) | 0.16±0.08 | 0.20±0.11 | ＜0.001 |
| Ceramide(d18:1/18:0)/ Ceramide(d18:1/24:0) | 0.05±0.03 | 0.06±0.04 | ＜0.001 |
| Ceramide(d18:1/24:1)/ Ceramide(d18:1/24:0) | 0.52±0.49 | 0.61±0.34 | ＜0.001 |

*P values of <0.05 were significant. P values were calculated according to the natural logarithm of the original value.

**Supplementary Table 2. Risk of combined events by baseline ceramide lengths and ratios**

| Model | CeramideType | HR | [95% Cl] | | P value |
| --- | --- | --- | --- | --- | --- |
| **Unadjusted** |  |  |  |  |  |
| Cer(d18:1/16:0) | | 1.95 | 1.39 -2.74 | | ＜0.001 |
| Cer(d18:1/18:0) | | 1.34 | 1.08 -1.65 | | 0.008 |
| Cer(d18:1/24:1) | | 1.33 | 1.02 -1.76 | | 0.029 |
| Cer(d18:1/24:0) | | 0.69 | 0.53 -0.91 | | 0.010 |
| Cer(d18:1/16:0)/Cer(d18:1/24:0) | | 2.10 | 1.62 -2.70 | | ＜0.001 |
| Cer(d18:1/18:0)/Cer(d18:1/24:0) | | 1.56 | 1.27 - 1.91 | | ＜0.001 |
| Cer(d18:1/24:1)/Cer(d18:1/24:0) | | 1.75 | 1.39 - 2.21 | | ＜0.001 |
| **Adjusted Model 1** | |  |  |  |  |
| Cer(d18:1/16:0) | | 1.89 | 1.35 - 2.68 | | ＜0.001 |
| Cer(d18:1/18:0) | | 1.33 | 1.08 - 1.65 | | 0.008 |
| Cer(d18:1/24:1) | | 1.34 | 1.03 - 1.75 | | 0.029 |
| Cer(d18:1/24:0) | | 0.66 | 0.50 - 0.88 | | 0.004 |
| Cer(d18:1/16:0)/Cer(d18:1/24:0) | | 2.15 | 1.65 - 2.80 | | ＜0.001 |
| Cer(d18:1/18:0)/Cer(d18:1/24:0) | | 1.58 | 1.29 - 1.93 | | ＜0.001 |
| Cer(d18:1/24:1)/Cer(d18:1/24:0) | | 1.83 | 1.44 - 2.32 | | ＜0.001 |
| **Adjusted Model 2** | |  |  |  |  |
| Cer(d18:1/16:0) | | 1.69 | 1.03 - 2.77 | | 0.037 |
| Cer(d18:1/18:0) | | 1.21 | 0.89 - 1.64 | | 0.236 |
| Cer(d18:1/24:1) | | 1.17 | 0.80 - 1.71 | | 0.420 |
| Cer(d18:1/24:0) | | 0.55 | 0.37 - 0.82 | | 0.003 |
| Cer(d18:1/16:0)/Cer(d18:1/24:0) | | 2.58 | 1.74 - 3.82 | | ＜0.001 |
| Cer(d18:1/18:0)/Cer(d18:1/24:0) | | 1.65 | 1.22 - 2.22 | | ＜0.001 |
| Cer(d18:1/24:1)/Cer(d18:1/24:0) | | 1.99 | 1.40 - 2.82 | | ＜0.001 |

Model 1 was adjusted for sex, age;

Model 2 was adjusted for sex, age, body mass index, systolic blood pressure, diastolic blood pressure, heart rate, diabetes, hypertension, hyperlipidemia, chronic renal disease, atrial fibrillation, coronary heart disease, and smoking. Effect size (OR and 95% CI) is presented for each ceramide length and ratio.

**Supplementary Figure 3. Graded relationship between number of dysfunctional/injured organs and survival in patients with worsening heart failure**

**
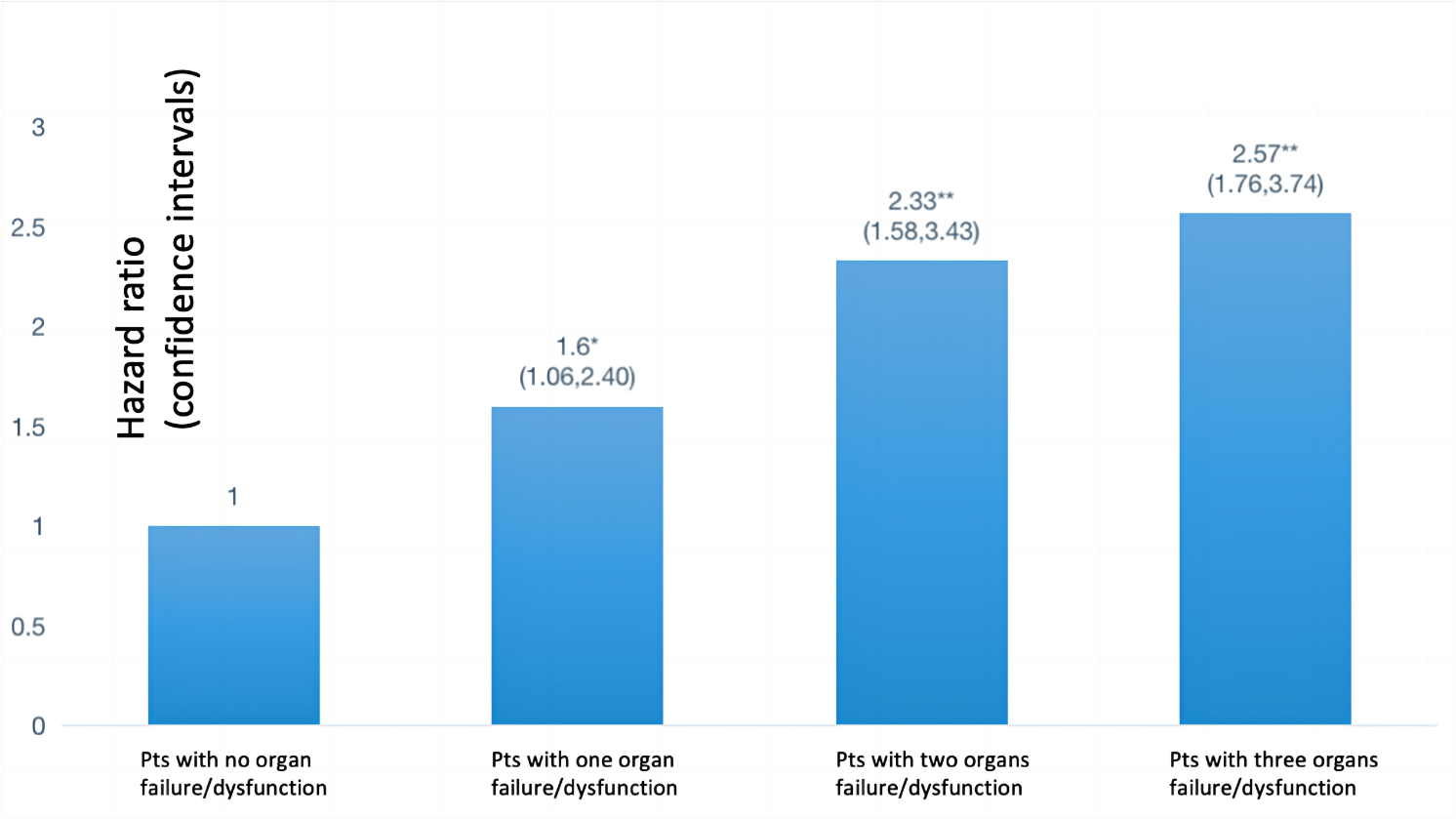
**

**Supplementary Table 3. Cox regression analysis for the CHFS and all-cause mortality and the combined endpoint**

| Feature | Univariable HR (95% CI) | P-value | Multivariable HR (95% CI) | P-value |
| --- | --- | --- | --- | --- |
| *Composite Event* | | | |  |
| CHFS in Discovery cohort | 2.80 (1.78-4.40) | ＜0.001 | 2.76(1.75-4.33) | ＜0.001 |
| CHFS in Validation cohort | 2.68 (1.12-6.46) | 0.028 | 3.47(1.36-8.87) | 0.009 |
| *All cause motility* | | | |  |
| CHFS in Discovery cohort | 3.07 (1.76-5.35) | ＜0.001 | 2.91(1.23-6.88) | 0.015 |
| CHFS in Validation cohort | 2.34 (1.10-6.75) | 0.016 | 3.45(1.10-10.80) | 0.034 |

The CHFS comprised the ceramide heart, liver, and kidney scores.

Multivariable HR model adjusted for sex, age for all-cause mortality and combined endpoint.

CHFS, ceramide heart failure score; CI, confidence interval; HR, hazard ratio; Ref, reference

**Supplementary Table 4. Characteristics of Validation cohort.**

| Clinical characteristics | No event group (N=240) | Event group (N=56) | P value |
| --- | --- | --- | --- |
| Male sex, n (%) | 147(61) | 42(75) | 0.054 |
| Age,yrs | 65±14.8 | 67±15.6 | 0.266 |
| **Clinical history, n (%)** | |  |  |
| Smoking | 38(23) | 6(21) | 0.818 |
| Hypertention | 140(63) | 27(56) | 0.420 |
| Diabetes mellitus | 15(33) | 75(35) | 0.721 |
| Hyperlipidemia | 77(36) | 19(41) | 0.526 |
| CKD | 50(24) | 13(30) | 0.413 |
| Prior MI | 40(19) | 10(24)  ) | 0.499 |
| Prior Stroke | 29(14) | 3(7) | 0.201 |
| Atrial brillation | 69(32) | 14(33) | 0.890 |
| VHD | 81(38) | 20(49) | 0.206 |
| CAD | 141(64) | 29(59) | 0.519 |
| SBP mmHg | 126±25 | 120±20 | 0.078 |
| DBP mmHg | 76±16 | 71±17 | 0.022 |
| Heart Rate b.p.m. | 87±22 | 85±21 | 0.637 |
| LVEF, % | 43±15 | 40±16 | 0.128 |
| **Laboratory Data** |  |  |  |
| HDLc, mmol/l | 1.01±0.31 | 0.95±0.29 | 0.633 |
| LDLc, mmol/l | 2.52±0.79 | 2.51±0.87 | 0.965 |
| TG, mmol/l | 1.30±0.78 | 1.34±0.91 | 0.841 |
| TC, mmol/l | 4.08±0.95 | 4.09±1.06 | 0.963 |
| hs-CRP | 9.08±9.34 | 14.69±10.76 | 0.052 |
| BNP | 1036±1204 | 1597±1321 | <0.001 |
| D-Dimer | 547.4±844.3 | 1474.7±3254.7 | <0.001 |
| **Therapy** |  |  |  |
| Statin | 91(50) | 11(38) | 0.217 |
| ACEI/ARB | 56(31) | 11(38) | 0.490 |
| β-blocker | 111(60) | 15(52) | 0.381 |
| Diuretic | 103(55) | 20(69) | 0.160 |
| Spironolactone | 81(45) | 15(52) | 0.468 |
| Digoxin | 45(25) | 5(18) | 0.411 |
| ANRI | 39(23) | 4(15) | 0.363 |

**Supplementary Table 5. Subgroup analysis of HFrEF, HFmrEF, and HFpEF in total population.**

| **Subgroup** | **Univariable** | | |  | **Multivariable** |  |  |
| --- | --- | --- | --- | --- | --- | --- | --- |
| **HFrEF(N=528)** | **Hazard Ratio (95% CI)** | **z-value** | **P value** |  | **Hazard Ratio (95% CI)** | **z-value** | **P value** |
| CHFS | 2.79(1.54-5.04) | 3.40 | 0.001 |  | 1.72(0.64-4.68) | 1.08 | 0.283 |
| **HFmrEF(N=195)** | **Hazard Ratio (95% CI)** | **z-value** | **P value** |  | **Hazard Ratio (95% CI)** | **z-value** | **P value** |
| CHFS | 2.97(1.00-8.80) | 1.97 | 0.049 |  | 3.11(0.43-22.44) | 1.13 | 0.260 |
| **HFpEF(N=509)** | **Hazard Ratio (95% CI)** | **z-value** | **P value** |  | **Hazard Ratio (95% CI)** | **z-value** | **P value** |
| CHFS | 2.63(1.36-5.12) | 2.86 | 0.004 |  | 3.85(1.50-9.87) | 2.81 | 0.005 |

Multivariable HR model adjusted for sex, age, body mass index, systolic blood pressure, diastolic blood pressure, heart rate, diabetes, hypertension, hyperlipidemia, chronic renal disease, atrial fibrillation, coronary heart disease, and smoking. Effect size (HR and 95% CI) is presented for ceramide heart failure score.

CHFS: Ceramide heart failure score; HFrEF: Heart failure with reduced ejection fraction; HFmrEF: Heart failure with middle range ejection fraction; HFpEF：Heart failure with preserved ejection fraction; HR, hazard ratio; CI, confidence interval

**Supplementary Table 6 Multicollinearity statistics of CHFS variables.**

| **CHFS variables** | **Hosmer-Lemeshow Chi^2^** | **P value** |
| --- | --- | --- |
| **Ceramide heart score** | 12.69 | 0.123 |
| **Ceramide liver score** | 3.98 | 0.859 |
| **Ceramide kidney score** | 12.35 | 0.136 |

**Supplementary Table 7 Goodness of fit of ceramide heart, liver, and kidney score in discovery set.**

| **Ceramide organ scores** | **Pseudo R^2^** | **LR test** | **Statistic** |  |
| --- | --- | --- | --- | --- |
| **Ceramide heart score** | 0.0163 | LR test: chibar2 = 17.39 (p<0.001) | BIC | 1060.305 |
|  |  |  | AIC | 1050.563 |
| **Ceramide liver score** | 0.0484 | LR test: chibar2 = 51.51 (p<0.001) | BIC | 1238.651 |
|  |  |  | AIC | 1228.995 |
| **Ceramide kidney score** | 0.0232 | LR test: chibar2 = 23.78 (p<0.001) | BIC | 1015.945 |
|  |  |  | AIC | 1006.629 |
|  |  |  | AIC | 1050.563 |

Ceramide heart score consisted of Cer(d18:1/18:0) and Cer(d18:1/18:0/ Cer(d18:1/18:0);

Ceramide liver score consisted of Cer(d18:1/16:0) and Cer(d18:1/24:1/ Cer(d18:1/24:0);

Ceramide kidney score consisted of Cer(d18:1/16:0) and Cer(d18:1/16:0/ Cer(d18:1/24:0).

AIC, Akaike information criteria; BIC, Bayesian information criteria;

**Supplementary Table 8 Multicollinearity statistics of CHFS variables.**

| **CHFS variables** | **Tolerance** | **VIF** |
| --- | --- | --- |
| **Ceramide heart score** |  |  |
| Cer(d18:1/18:0) | 0.456 | 2.19 |
| Cer(d18:1/18:0/ Cer(d18:1/18:0) | 0.456 | 2.19 |
| **Ceramide liver score** |  |  |
| Cer(d18:1/16:0) | 0.938 | 1.07 |
| Cer(d18:1/24:1/ Cer(d18:1/24:0) | 0.938 | 1.07 |
| **Ceramide kidney score** |  |  |
| Cer(d18:1/16:0) | 0.818 | 1.22 |
| Cer(d18:1/16:0/ Cer(d18:1/24:0) | 0.818 | 1.22 |

VIF, Variance Inflation Factor

**Supplementary Table 9 Global test for proportional-hazards assumption in discovery and validation set.**

| **Feature** | **C-index** | **Chi^2^** | **df** | **Prob＞chi^2^** |
| --- | --- | --- | --- | --- |
| **CHFS in Discovery set** | 0.60(0.56,0.64) | 0.95 | 1 | 0.329 |
| **CHFS in Validation set** | 0.59(0.51,0.67) | 1.25 | 1 | 0.264 |

**Supplementary Figure 4 Result of logistic regression analysis testing association between ceramide heart, liver, kidney score and clinical status.**


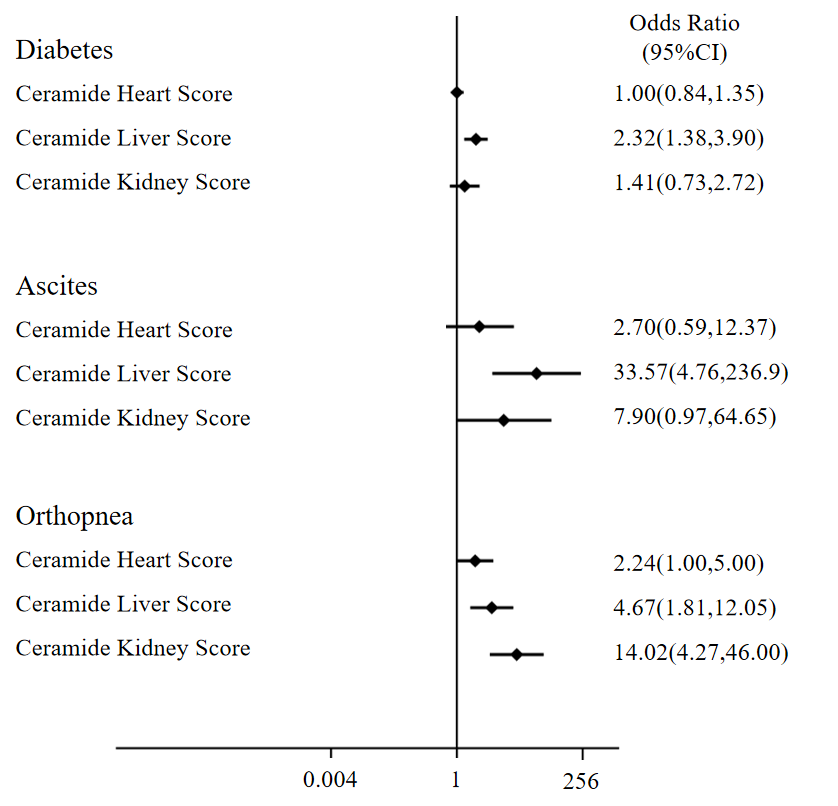


**Supplementary Table 10 Formula of Logistic Regression Models and Ceramide Heart Failure Score.**

| **Ceramide scores** | **Formula** |
| --- | --- |
| **Ceramide heart score** | Log (Odds) = 0.9544444×Cer(d18:1/18:0) ^*^- 0.1710524× Cer(d18:1/18:0)/Cer (d18:1/24:0) ^*^-4.658546 |
| **Ceramide Liver score** | Log (Odds) = 0.6787771×Cer(d18:1/16:0) ^*^+0.779046× Cer(d18:1/24:1)/Cer (d18:1/24:0) ^*^-3.100206 |
| **Ceramide Kidney score** | Log (Odds) =0.2077131×Cer(d18:1/16:0) ^*^+0.0107772× Cer(d18:1/16:0)/Cer(d18:1/24:0) ^*^-0.6739453 |
| **Ceramide Heart Failure Score** | CHFS = Ceramide heart score + Ceramide liver score + Ceramide kidney score |

* For ceramide length and ratio variables that were natural log trans-formed.
